# Letter to the Editor: A commentary on ‘Successful robotic kidney transplantation for surgeons with no experience in minimally invasive surgery: a single institution experience’

**DOI:** 10.1097/JS9.0000000000001751

**Published:** 2024-05-29

**Authors:** Yiqi Huang, Yanling Zhang, Sanmeizi Xie

**Affiliations:** a Department of Nephrology, Shaoxing Second Hospital; b Intensive Care Unit, Shaoxing Second Hospital, Shaoxing, Zhejiang, People’s Republic of China

*Dear Editor,*

Kidney transplantation (KT) is the best choice for patients with end-stage renal disease (ESRD), and open kidney transplantation (OKT) is the current mainstream surgical method, but it has many complications such as large trauma and long postoperative recovery time^1^. The world’s first robotic-assisted laparoscopic kidney transplant (RKT) was successfully conducted by Professor Giulianotti and his team from the United States in 2010^2^. Clinicians have gradually embraced its safety and reliability over more than a decade of development. However, RKT requires a high level of technical skill from the operating doctor, so it has not yet been widely used in clinical practice. The retrospective cohort study conducted by Kim *et al*.^3^ examined successful robotic KT for surgeons with no experience in minimally invasive surgery (MIS), and findings indicated that both the RKT and OKT groups exhibited similar intraoperative and postoperative outcomes as well as renal function^3^. However, the RKT group experienced a prolonged surgical duration compared to the OKT group, while significant reductions in estimated blood loss and postoperative hospital stays were demonstrated. Furthermore, it was noted that a single surgeon achieved surgical competence in RKT after 15 cases.

Nevertheless, it is crucial to recognize three major limitations of this study. Firstly, Kim *et al*.^3^ concentrated primarily on the analysis of perioperative complications in both the RKT and OKT groups, noting similar recovery of renal function during this period. However, they did not comprehensively investigate chronic complications related to KT, specifically alterations in renal function from 3 to 6 months postoperatively. Therefore, we recommend that Dr Kim prolong the observation and follow-up period.

Secondly, to prevent kidney dysfunction during rewarming, Dr Kyu Ha Huh employed the conventional local hypothermia technique of RKT^3^. This technique entails enveloping the transplanted kidney with gauze containing icy slush during vascular anastomosis and intermittently adding icy slush to ensure continuous cooling of the transplanted kidney within the abdominal cavity^4^. The drawback of this method lies in the susceptibility of the gauze with icy slush to rapid melting, necessitating a constant addition of supplementary icy slush around the kidney throughout the procedure, thereby impeding operational continuity. Simultaneously, the liquefied icy slush will permeate through the gauze and enter the abdominal cavity, increasing the patient’s risk of postoperative intestinal obstruction. Tugcu *et al*.’s^5^ study demonstrated that 2 out of 15 patients who underwent RKT experienced postoperative anesthetic ileus due to the addition of icy slush. Based on this, the author made a theoretical improvement to the technique and suggested the utilization of a double-layer plastic bag for enveloping the transplanted kidney, with icy slush occupying the outer layer. The main advantages of this approach are two-fold: the icy slush fills the space between the two plastic bags, and the insulating effect of the plastic bag reduces heat exchange between the kidney and its surroundings; even if some of the icy slush melts, they will form a mixture of ice and water that continues to surround the kidney, without spreading into the abdomen, keeping the kidney at a relatively constant low temperature and preventing anesthetic ileus and other complications to a great extent.

Finally, Dr Kyu Ha Huh, an expert in OKT with no prior experience in MIS, dedicated 12 months to completing the Da Vinci training program and an online training course before undertaking his inaugural RKT surgery^3^. Additionally, he honed his surgical skills for the robotic system through simulator-based training. This comprehensive training model extended the duration of RKT surgeon preparation. Consequently, expediting and optimizing the training process for qualified RKT surgeons emerges as a pivotal objective in further advancing RKT surgery. We propose exploring the possibility of selecting experienced urologists with extensive expertise in MIS for RKT training, which could potentially yield positive outcomes by reducing the duration of training and conserving medical resources.

Overall, the findings of this retrospective study are commendable as they confirm that, through adequate preparation including essential training, even surgeons lacking experience in MIS can proficiently acquire fundamental robotic surgery techniques and safely perform RKT. Consequently, we strongly recommend that teams possessing extensive expertise in OKT surgery and minimally invasive procedures consider undertaking this procedure.

## Ethical approval

Not applicable.

## Consent

Not applicable.

## Source of funding

Not applicable.

## Author contribution

Y.H., Y.Z., and S.X.: conceptualization, methodology, validation, formal analysis, investigation, resources, data curation, writing – original draft, writing – review and editing, supervision, and project administration.

## Conflicts of interest disclosure

The authors declare that there are no conflicts of interest regarding the publication of this paper.

## Research registration unique identifying number (UIN)

Not applicable.

## Guarantor

Yiqi Huang, Yanling Zhang, and Sanmeizi Xie.

## Data availability statement

Data sharing is not applicable to this article as no new data were created or analyzed in this study.

## Provenance and peer review

Not applicable.
